# Semen Cryopreservation and Semen Quality in Transfeminine Adolescents Prior to Hormone Therapy

**DOI:** 10.1210/jendso/bvaf105

**Published:** 2025-06-10

**Authors:** Mohsin Aslam, Pernille Badsberg Norup, Mette Ewers Haahr, Anne Katrine Pagsberg, Annamaria Giraldi, Niels Jørgensen, Katharina Maria Main

**Affiliations:** Department of Growth and Reproduction, Copenhagen University Hospital—Rigshospitalet, 2100 Copenhagen, Denmark; International Centre for Research and Research Training in Endocrine Disruption of Male Reproduction and Child Health (EDMaRC), Rigshospitalet and University of Copenhagen, 2100 Copenhagen, Denmark; Department of Growth and Reproduction, Copenhagen University Hospital—Rigshospitalet, 2100 Copenhagen, Denmark; International Centre for Research and Research Training in Endocrine Disruption of Male Reproduction and Child Health (EDMaRC), Rigshospitalet and University of Copenhagen, 2100 Copenhagen, Denmark; Sexological Clinic, Mental Health Centre Copenhagen, Copenhagen University Hospital, Mental Health Services—Capital Region of Denmark, 2200 Copenhagen, Denmark; Department of Clinical Medicine, University of Copenhagen, 2200 Copenhagen, Denmark; Department of Clinical Medicine, University of Copenhagen, 2200 Copenhagen, Denmark; Child and Adolescent Mental Health Centre, Copenhagen University Hospital, Mental Health Services—Capital Region of Denmark, 2900 Hellerup, Denmark; Sexological Clinic, Mental Health Centre Copenhagen, Copenhagen University Hospital, Mental Health Services—Capital Region of Denmark, 2200 Copenhagen, Denmark; Department of Clinical Medicine, University of Copenhagen, 2200 Copenhagen, Denmark; Department of Growth and Reproduction, Copenhagen University Hospital—Rigshospitalet, 2100 Copenhagen, Denmark; International Centre for Research and Research Training in Endocrine Disruption of Male Reproduction and Child Health (EDMaRC), Rigshospitalet and University of Copenhagen, 2100 Copenhagen, Denmark; Department of Growth and Reproduction, Copenhagen University Hospital—Rigshospitalet, 2100 Copenhagen, Denmark; International Centre for Research and Research Training in Endocrine Disruption of Male Reproduction and Child Health (EDMaRC), Rigshospitalet and University of Copenhagen, 2100 Copenhagen, Denmark; Department of Clinical Medicine, University of Copenhagen, 2200 Copenhagen, Denmark

**Keywords:** semen quality, fertility preservation, reproductive health, transgender health

## Abstract

**Background:**

Some transgender individuals wish to become biological parents in the future and transfeminine persons may be offered cryopreservation of semen before starting hormone therapy. However, there is a lack of knowledge about semen cryopreservation outcomes among transfeminine adolescents seeking gender-affirming care.

**Objective:**

To investigate transfeminine adolescents who decide for or against semen cryopreservation.

**Methods:**

This is a retrospective observational national cohort study of 58 transfeminine individuals aged <18 years, assessing clinical data, semen parameters, and reproductive hormone levels.

**Results:**

Among the 58 individuals, 23 (39.7%) opted for semen cryopreservation and successfully collected a semen sample. They were older and more advanced in pubertal development compared with those who did not: median age was 16.4 years (range, 13.7-19.4) vs 15.8 years (11.7-17.9); Tanner stage G5 (4-5) vs G3 (2-4); and testis volume 20 mL (15-25) vs 8 mL (3-20). Among 17 individuals with no prior hormone therapy, the median sperm concentration was 11.1 × 10^6^/mL (0.02-163), semen volume 1.8 mL (0.2-3.9), total sperm count 17.8 × 10^6^ (0.1-214.2), and percentage of progressively motile spermatozoa 46% (8-74). Reproductive hormones were within normal ranges for age and registered sex at birth.

**Conclusion:**

The percentage of adolescents opting for semen cryopreservation was comparable to other countries with a publicly financed national healthcare system. Overall, semen quality was impaired.

There has been an increase in young individuals with gender incongruence seeking health care and gender-affirming hormone therapy (GAHT) worldwide [1-4] as well as in Denmark [5].

Transgender individuals experience gender incongruence, that is, the registered sex at birth differs from their gender identity. In this study, transfeminine individuals represent those registered male at birth who identify as female. Current knowledge of long-term reproductive health and the demand for fertility preservation among transgender and gender-diverse individuals under the age of 18 years is limited.

Current clinical guidelines recommend informing transgender individuals about the reproductive adverse effects, including the potential loss of fertility when undergoing hormone therapy and providing information about fertility preservation options [6, 7]. Hormone therapy may be commenced once puberty has started and the indication has been established. For transfeminine adolescents, endocrine treatment comprises puberty-suppressing gonadotropin-releasing hormone agonists (GnRHa) which inhibit gonadotropins, luteinizing hormone (LH), and follicle-stimulating hormone (FSH), and consequently testis growth and development, testosterone secretion, and spermatogenesis [7-9]. Estrogen is administered for developing feminine characteristics, typically from the age of 15 to 16 years. Estrogen treatment combined with antiandrogen treatment has suppressive effects on male reproductive function and reduces semen quality [10, 11].

Some transgender individuals wish to have biological children, and some express regret over missed chances for fertility preservation [12, 13]. However, fertility preservation rates seem to be low among transgender youth possibly due to psychological barriers, such as severe gender dysphoria and the need to discontinue or delay starting GAHT [14, 15], and physical barriers, including the youngster's stage of pubertal development and not yet being able to produce a semen sample [16].

Few studies have investigated semen quality in transfeminine youth. Overall, semen quality appears to be lower than that of reference groups registered male at birth [17-22]. Thus, studies of frequencies of semen cryopreservation and semen quality in young transfeminine individuals under the age of 18 remain limited [14, 23, 24]. In Denmark, semen cryopreservation is offered free of charge in the public health care system to transfeminine individuals, if they can produce a semen sample through masturbation before initiating any hormone treatments. This study examines the frequency of transfeminine individuals referred for hormone therapy opting for or against collecting semen samples for cryopreservation. It assesses semen quality and reproductive hormone concentrations.

## Methods

The Department of Growth and Reproduction, Rigshospitalet, Capital Region, Denmark, serves as the only public tertiary nationwide center for GAHT for all transgender adolescents aged <18 years in Denmark.

### Study Design

This national, retrospective observational cohort study encompasses transgender individuals who were evaluated and treated within the National Health Care System before the age of 18 years. Clinical data were retrieved from medical records of transfeminine adolescents referred to the Department of Growth and Reproduction for hormone therapy between January 2016 and December 2022.

### Ethics

This study was registered at the Capital Region of Denmark (P-2019-230) and received approval by the Local Ethics Committee (H-18050607). The Danish Patient Safety Authority (3-3013-3117/1) and the Centre for Health in the Capital Region's team for Medical Records Research (R-23014626) granted authorization to access medical records between January 1, 2016, and December 31, 2022, with a waiver of informed consent.

### Study Population

A total of 58 individuals were referred for hormone therapy consisting of GnRHa only or GnRHa combined with estradiol. The treatments for this study population were described in an earlier publication [25]. Before initiating hormone therapy, the individuals underwent a comprehensive evaluation by a multidisciplinary team of psychologists, child and adolescent psychiatrists, and pediatric endocrinologists [5]. Adolescents and their caregivers received information about the known and potential effects, side effects, and risks of GAHT, including the impact on future reproductive health and fertility. Semen cryopreservation was offered to transfeminine individuals according to current guidelines if biologically possible [6, 7, 26].

### Data Retrieval

Data from clinical examinations were retrieved from medical records: age, pubertal development according to Tanner, testis volume, results from semen analyses, and reproductive serum hormone concentrations. Body mass index (BMI) and BMI standard deviation scores (SDS) for age and registered sex at birth were retrieved from GrowthXP (PC PAL, Bièvres, France) based on weight and height. BMI SDS were based on a Danish reference population [27]. Tanner stage, testicular volume, and BMI were included if measured no more than 3 months before the initiation of hormone therapy. If the Tanner stage G5 was found earlier, this was included.

Tanner stage and testicular volume were assessed in the youngest individuals only to confirm the stage of puberty, following the evaluation of growth curves, current height, age, and visual appearances of secondary sexual characteristics [28, 29]. The initiation of GAHT required a Tanner stage of G2 or higher. The testis volume was assessed with Prader's orchidometer.

### Semen Collection and Analyses

Semen samples were obtained by masturbation and ejaculated into clean wide-mouthed plastic containers in a private room near the semen laboratory at the department. The individuals were instructed to abstain from ejaculation for at least 48 hours before sampling. Self-reported period of abstinence was recorded along with information concerning episodes of fever within the last 3 months, previous cryptorchidism, varicocele, trauma, infection, or surgery related to the urogenital system. Depending on the quality of the first semen sample, it was decided whether additional collections would be offered.

The semen sample was kept at 37 °C during liquefaction. Semen volume (mL) was determined by weighing the collection containers, assuming 1 g to be equivalent to 1 mL. Sperm concentration (×10^6^/mL) was determined using a Bürker-Türk hemocytometer and total sperm count was calculated (semen volume × sperm concentration). To assess sperm motility, 2 drops of well-mixed semen were placed on a glass slide and examined with phase contrast–microscopy, classifying the spermatozoa as progressive motile, non-progressive motile, and immotile. The total motility was defined by subtracting the immotile class percentage from 100%. Assessments were done in accordance with the World Health Organization (WHO) guidelines [30]. Azoospermia was reported if no spermatozoa were found under these evaluations. All analyses are accredited by the Danish Accreditation Fund (DANAK, www.danak.dk). All expenses including those of cryopreservation and semen analysis were covered by the public healthcare system. Individuals could also opt for semen cryopreservation at private clinics due to long travel distances.

### Hormone Analyses

Non-fasting blood samples were drawn from an antecubital vein either at the time of or up to 5.3 months before, the collection of the first semen sample or before treatment, depending on whether participants opted for or opted out of cryopreservation.

Serum FSH, LH, total testosterone, inhibin-B, and sex-hormone-binding globulin were measured in the department's laboratory which is accredited for all analyses (DANAK, www.danak.dk). One case of nonclassical congenital adrenal hyperplasia was identified during the initial endocrine evaluation [31] and excluded from hormone analyses. Hormone concentrations are presented as absolute values and SDS for age and registered sex at birth compared to local references [32-34]. FSH and LH serum concentrations were measured using 2 different assays over the study period. The first method used time-resolved immunofluorometric assays (AutoDelfia; PerkinElmer, MA, USA) (*n* = 43) with limits of detection (LOD) of 0.05 IU/L and inter-assay coefficients of variation (CVs) below 9%. The second method used chemiluminescence immunoassays (Atellica; Siemens Healthineers, Tarrytown, NY, USA) (*n* = 1) with LOD of 0.3 IU/L and 0.07 IU/L, respectively, and CVs ≤ 20% and ≤ 30%, respectively. FSH serum concentrations from the immunofluorometric method were converted to equivalent serum concentrations from the chemiluminometric method using an internal correction factor adjusting for instrument bias. No correction was necessary for LH measures between the 2 methods applied.

Total testosterone serum concentrations were measured by liquid chromatography–tandem mass spectrometry (LC-MS/MS) with a LOD of 0.1 nmol/L and CVs ≤ 10% (*n* = 44). Estradiol serum concentrations were measured either by radioimmunoassay (Pantex, USA) (*n* = 18) with LOD of 18.1 pmol/L and CVs ≤ 15% or LC-MS/MS (*n* = 25) with a LOD of 12.1 pmol/L and CVs ≤ 13%. Estradiol serum concentrations measured by radioimmunoassay were converted to equivalent serum concentrations from LC-MS/MS using an internal correction factor adjusting for instrument bias. Inhibin-B serum concentrations were measured by an enzyme-linked immunosorbent assay (ELISA) (Catalog # A81303, RRID: AB_2827405) (*n* = 44) with a LOD of 3 ng/L and a CV ≤ 12%. A bivariate plot of inhibin-B vs FSH levels was depicted with a 97th percentile curve as a reference line based on 276 fathers aged 20 to 45 years with a sperm concentration above 20 × 10^6^/mL [35]. Measurements below LOD were assigned the value of LOD/2. Free testosterone levels (*n* = 44) were calculated using the equation described by Vermeulen et al based on the concentrations of total testosterone and sex-hormone-binding globulin while assuming a fixed albumin concentration of 43.0 g/L [36].

### Statistics

Unless indicated otherwise, descriptive statistics are presented as median and range (min-max). If multiple semen samples were collected from an individual, the first sample was chosen for statistical analyses. Repetitive samples are depicted visually in figures. Group differences between individuals who did and did not attempt semen cryopreservation were tested by Mann-Whitney U test.

Data from cases reporting self-medication, prior GAHT, or congenital adrenal hyperplasia, or individuals opting for semen cryopreservation at private clinics were excluded from analyses, tables, and figures of semen parameters and hormone levels.

Semen parameters were compared to the cutoff values of 2 existing reference publications: Cutoff values from WHO 5th percentiles are semen volume of 1.4 mL, sperm concentration of 16 × 10^6^/mL, total sperm count of 39 × 10^6^, total motility percentage of 42%, progressive motility of 30%, non-progressive motility of 1%, and immotile spermatozoa percentage of 20% [30]. Cutoff values defined from studies examining associations between semen parameters and the chance of conception through intercourse are set to a concentration of 40 × 10^6^/mL [37] and a total motility percentage of 32% [38]. A *P* value <.05 was considered statistically significant. Statistical analyses were conducted using R version 4.2.2 [39].

## Results

### Population Characteristics

A total of 58 transfeminine adolescents were referred for the start of GnRHa or GAHT at the median age of 15.9 years (11.4-19.3). Semen collection was successful in all 23 (39.7%) who opted for semen cryopreservation and 22 had at least one semen sample cryopreserved. Those who delivered a semen sample were significantly older than those who did not: median age of 16.4 years (13.7-19.4) vs 15.8 years (11.7-17.9) (Table 1). They were also more advanced in pubertal development and had a larger testis volume. Of the 35 individuals who did not attempt semen sample collection, 32 opted out of semen cryopreservation and the status of 3 was unknown. In both groups, 3 individuals had a history of prior GAHT or self-medication.

**Table 1.** Population characteristics

	Opting for semen cryopreservation			Opting out of semen cryopreservation			*P* value*^a^*
	*n*	median	(range)	*n*	median	(range)	
Age at first counseling on semen cryopreservation (years)	23	16.4	(13.7-19.4)	32	15.8	(11.7-17.9)	< .05
Age at initiation of hormone therapy (years)	22	16.8	(14.1-19.5)	32	15.9	(12.0-17.9)	< .05
Tanner stage*^b^* (G)	5	5.0	(4-5)	9	3	(2-4)	< .05
Left testis volume (mL)	6	20.0	(15-25)	9	8	(3-20)	< .05
Right testis volume (mL)	6	20.0	(15-25)	9	8	(4-20)	< .05
BMI*^c^*	20	20.0	(15.0-31.3)	31	19.9	(16.0-37.0)	.83
BMI SDS*^d^*	20	−0.4	(−3.2-2.6)	31	−0.1	(−1.1-4.1)	.24
Self-medication or prior GAHT*^e^*	3	NA	NA	3	NA	NA	NA
Collected a semen sample at the department	20	NA	NA	NA	NA	NA	NA
Collected a sample at a private sperm bank	3	NA	NA	NA	NA	NA	NA

^a^ *P* value: Mann-Whitney U test.

^b^Clinical assessment a.m. Tanner [29].

^c^Body mass index.

^d^Body mass index standard deviation scores for age and registered sex at birth in a Danish reference population [27].

^e^Gender-affirming hormone therapy.

### Semen Parameters

**Figure 1.**
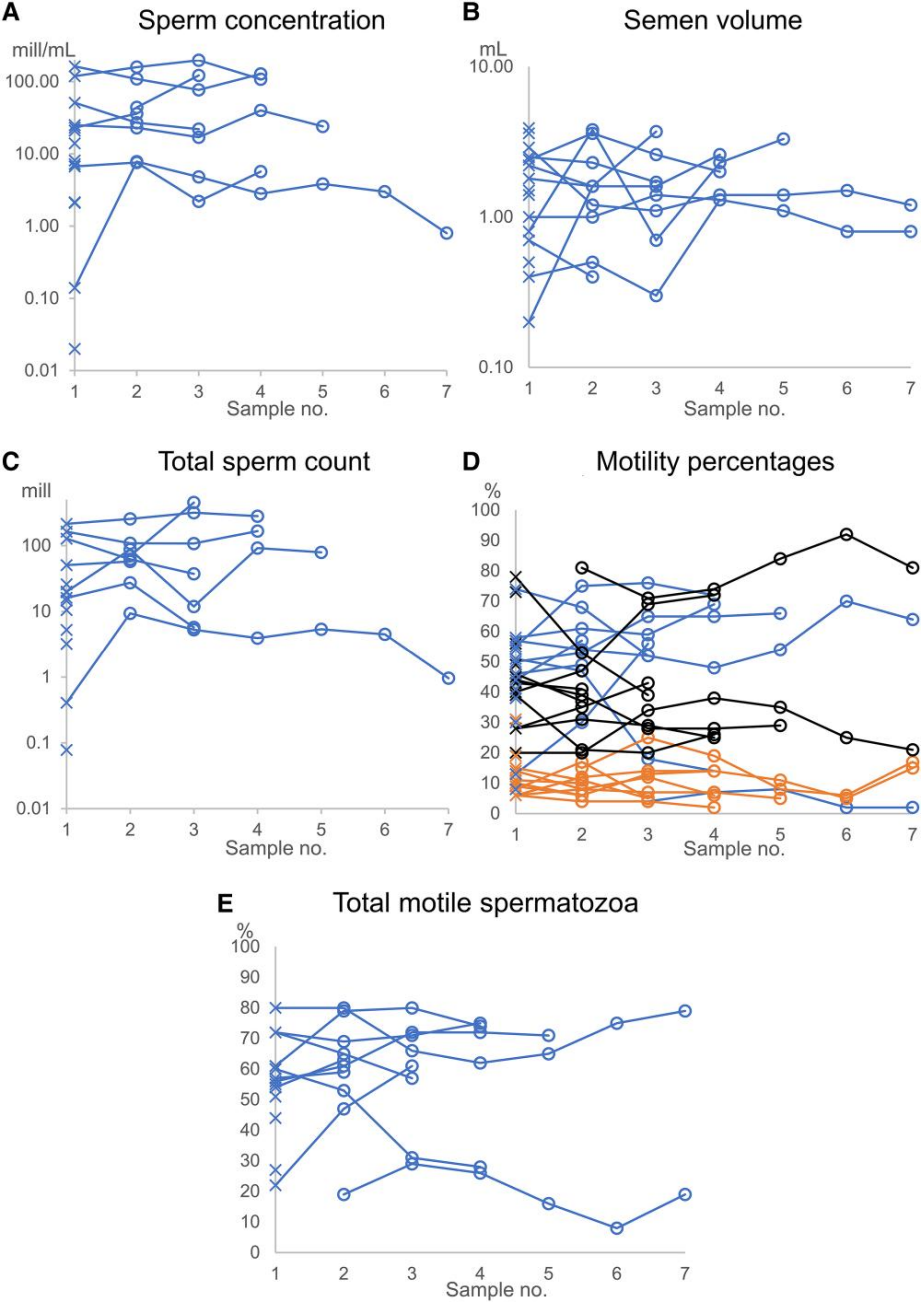
Semen parameters prior to semen collection shown as repetitive samples in chronological order. Each line or single cross represents one individual with no history of self-medication or hormone therapy. Crosses (**×**) indicate the initial sample. A) Sperm concentration (×10^6^/mL). B) Semen volume (mL). C) Total sperm count (×10^6^). D) Motility class percentages: blue (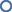) progressive motile spermatozoa, orange (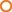) non-progressive motile spermatozoa, black (○) immotile spermatozoa. E) Total motile spermatozoa.

A total of 17 individuals, who had not received prior GAHT or self-medication, collected at least one semen sample at the department, while 3 others chose private sperm banks for cryopreservation. This made 17 individuals eligible for semen parameter analysis (Table 2). Of these 17 individuals, 3 had a semen volume too small to determine the sperm concentration; however, they had spermatozoa in their ejaculates. The remaining 14 had a median sperm concentration of 11 × 10^6^/mL, corresponding to 57% having a concentration lower than 16 × 10^6^/mL and 79% lower than 40 × 10^6^/mL (Supplementary Table S1 [40]). The median total sperm count was 18 × 10^6^, corresponding to 71% having a total sperm count lower than 39 × 10^6^. The median percentage of progressive motile spermatozoa was 46%, corresponding to 13% having a percentage of progressive motile lower than 30%. The total percentage of motile spermatozoa was 57%, corresponding to 13% having a total motile percentage lower than both cutoffs set to 42% and 32%. Median abstinence time was 2.5 days (0-7, *n* = 10). Available data (*n* = 7) showed no history of fever in the past 3 months, cryptorchidism, varicocele, known fertility problems, infection, surgery, or injury related to the urogenital system. Overall, semen quality did not vary considerably in individuals who provided more than one semen sample (Fig. 1).

**Table 2.** Semen parameters

	Semen samples collected at the department (*n* = 17)		
	*n*	median	(range)
Age at sample collection (years)	17	16.8	(14.1-19.5)
Samples per person	17	2.0	(1-7)
Semen volume (mL)	17	1.8	(0.2-3.9)
Sperm concentration (10^6^/mL)	14	11.1	(0.02-163.00)
Total sperm count (10^6^)	14	17.8	(0.1-214.2)
Progressive motile spermatozoa (%)	15	46.0	(8.0-74.0)
Non-progressive motile spermatozoa (%)	15	9.0	(6.0-31.0)
Immotile spermatozoa (%)	15	43.0	(20.0-78.0)
Total motile spermatozoa (%)	15	57.0	(22.0-80.0)
Samples with azoospermia	0	NA	NA

Semen parameters of the initial semen sample for each individual with no history of prior self-medication or gender-affirming hormone therapy.

### Reproductive Hormones

**Figure 2.**
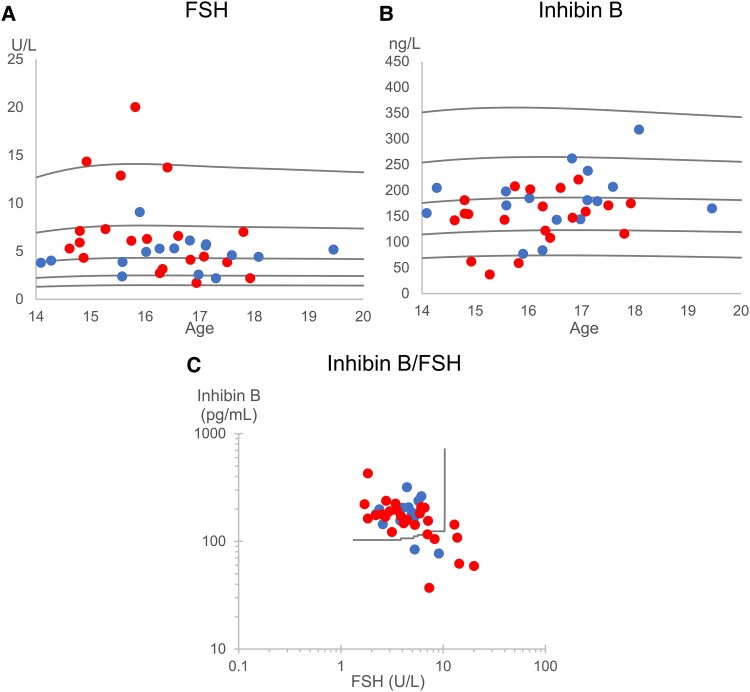
Reproductive hormones related to Sertoli cell function shown against age and sex-adjusted references. Reference lines (black) represent mean, ±1 SD, and ±2 SD scores. A) FSH, B) Inhibin B, C) Bivariate plot of inhibin B vs FSH with 97th percentile curve as reference line [35]. Blue dots (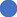): Individuals collecting a semen sample; red dots (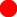): Individuals not opting for cryopreservation.

**Figure 3.**
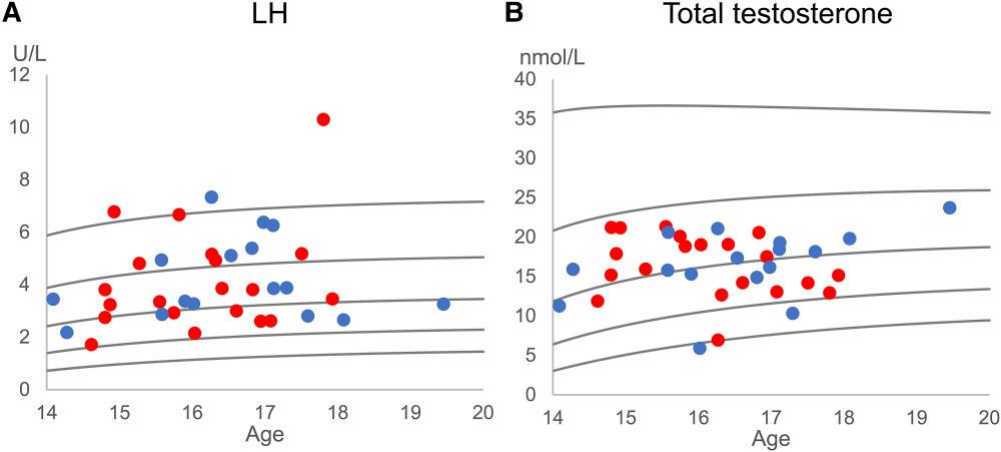
Reproductive hormones related to Leydig cell function shown against age and sex-adjusted references. Reference lines (black) represent mean, ±1 SD, and ±2 SD scores. A) LH, B) Total testosterone. Blue dots (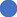): Individuals collecting a semen sample; red dots (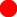): Individuals not opting for cryopreservation.

Overall, reproductive hormones were within the reference ranges for age and registered sex at birth (Table 3) for both Sertoli cell function (FSH and inhibin-B) (Fig. 2), and Leydig cell function (LH and total testosterone) (Fig. 3). There were no differences in serum concentrations between the group collecting semen samples and the group opting out of semen cryopreservation. However, despite being within normal ranges, estradiol SDS was significantly lower in those collecting a semen sample. In Fig. 2C, the inhibin-B/FSH ratio was outside the reference for 9 individuals.

**Table 3.** Serum concentrations of reproductive hormones before hormone therapy

	Collected semen samples at the department			Opting out of semen cryopreservation			*P* value*^a^*
	n	median	(range)	n	median	(range)	
**Concentrations**							
FSH (U/L)	16	4.8	(2.2-9.1)	28	4.4	(1.7-20.0)	.76
LH (U/L)	16	3.7	(2.2-7.3)	28	3.0	(1.2-10.3)	.06
Total testosterone (nmol/L)	16	16.7	(5.9-23.7)	28	14.2	(0.9-21.3)	.11
Inhibin-B (pg/mL)	16	180.0	(77-318)	28	166.0	(37.0-427.0)	.29
Free testosterone (pmol/L)	16	369.5	(119.2-590.3)	28	357.5	(7.8-469.6)	.09
Estradiol (pmol/L)	16	78.2	(31.4-125.6)	27	93.3	(15.7-127.0)	.82
**Standard deviation scores*^b^***							
FSH (SDS)	16	0.2	(−1.2-1.3)	28	0.5	(−1.7-2.6)	.25
LH (SDS)	16	0.4	(−0.5-2.2)	28	0.4	(−0.9-3.2)	.54
Total testosterone (SDS)	16	0.1	(−2.2-0.7)	28	0.1	(−2.0-1.7)	.85
Inhibin-B (SDS)	16	−0.1	(−1.9-1.6)	28	−0.2	(−3.1-3.0)	.54
Estradiol (SDS)	16	0.1	(−2.1-1.5)	27	0.8	(−2.1-2.2)	<.05

Individuals collecting a semen sample at the department compared with the group not opting for cryopreservation with no history of prior self-medication or gender-affirming hormone therapy. Concentrations and standard deviation scores (SDS) are depicted as medians (range).

^a^ *P* value: Mann-Whitney U test.

^b^SDS adjusted for age and registered sex at birth.

## Discussion

In this national cohort of transfeminine individuals aged 13.7 to 19.4 years, 39.7% of the individuals referred for hormone therapy chose to attempt semen cryopreservation. Although all individuals successfully collected one or more semen samples, the overall semen quality of those with no prior history of GAHT was reduced compared to WHO standards and the cutoff values. A considerable percentage of transfeminine adolescents opted out of semen cryopreservation, which underscores the complexity of addressing fertility wishes at a young age [6, 7]. Those who chose cryopreservation were older and more advanced in puberty than those who did not. This may reflect that semen cryopreservation was only offered to young individuals with a testis volume of at least 8 mL [41, 42]. It may also be due to being more mature to be able to consider future parenthood and to have more experience with masturbation.

In agreement with our findings, the use of fertility preservation has been described as low among transgender and gender-diverse individuals with only a few studies focusing on adolescents [14, 15]. An American study showed that 16.7% of 36 transfeminine adolescents and young adults (median age 15.7 years) underwent semen cryopreservation [43]. In the United Kingdom, 50% out of 24 transfeminine adolescents pursued the option of fertility preservation, but only 5 completed fertility preservation [44]. In Denmark, semen cryopreservation is free of charge in the National Health Care Service. Our findings resemble studies from other countries with a public health care system. In the Netherlands, 34.3% of 35 transfeminine adolescents (mean age 14.8 years) attempted cryopreservation [45]. An Australian study described that 62% of 53 transfeminine adolescents (mean age 15.6 years) pursued fertility preservation, and 42% successfully cryopreserved semen [46].

Our finding of an overall suboptimal semen quality among transfeminine adolescents is in line with previous studies. One study of 29 transfeminine adolescents and young adults (median age 28 years) found a median sperm concentration of 35 × 10^6^/mL, progressive motility of 45%, and 6% normal morphology [18]. Another study reported that transfeminine adolescents and young adults (median age 24.1 years) had lower sperm concentration (median 14.2 × 10^6^/mL), sperm count (55.7 × 10^6^), total motile sperm count (35.0 × 10^6^), higher rates of samples with less than 15 million spermatozoa per mL, and increased cryosensitivity, compared to cisgender controls [19]. All semen parameters in another population of young transfeminine individuals (mean age of 15.9 years) were lower than WHO references [17]. Another study found that semen quality in transfeminine adolescents (mean age 15.8 years) tended to be lower than in peers with cancer before gonadotoxic therapy; however, this difference was not statistically significant [47]. A recent large study from the United Kingdom of 78 transgender adolescents (mean age of 16.3 years) reported a higher mean sperm concentration (22.7 million/mL) and a total sperm count (34.2 million) than seen in our study population [20]. We have currently no explanation for this difference. When comparing semen parameters in our study with data from contemporary young Danish men of the general population (median age 19 years) [48], our study population exhibited suboptimal semen quality for all parameters. In addition, 57% had a sperm concentration lower than the WHO 5th percentile [30], and 79% lower than the threshold where the chance of achieving pregnancy by intercourse decreases [37].

Clinical guidelines indicate that the choice of Medically Assisted Reproduction (MAR) treatment, that is, intrauterine insemination (IUI), in vitro fertilization (IVF), or intracytoplasmic sperm injection (ICSI), depends on semen quality and quantity, including post-thaw characteristics [49]. Given the ranges of pre-freeze values in our study population, most samples would likely be suitable for use in MAR such as ICSI.

Studies of semen quality in adult trans women suggest that semen quality may be impaired due to behavioral and lifestyle factors, such as wearing tight undergarments and genital tucking [21, 50, 51]. We did not systematically collect this information. Reproductive hormone levels were within the age-adjusted reference range, which thus does not explain the low semen parameters observed and also does not support undisclosed self-medication. Stress may cause dysregulation of hypothalamic function, and thus pituitary hormones [52-54], but this was not found in our population. The inhibin-B/FSH ratio results suggest a primary spermatogenic reduction.

Transfeminine individuals face several barriers to accessing fertility preservation [45]. The first occurrence of sperm production, spermarche and spermaturia, happens late in puberty [16], thus presenting a biological restriction for individuals in early puberty. An early initiated treatment with GnRHa and sex steroids can prevent the progression of sexual characteristics and gender incongruence [9-11]. Therefore, transgender adolescents are faced with the decision to balance the advantages of early treatment against the potential negative impact on future options for biological parenthood. An individual can choose to pause GAHT; however, this may be unacceptable and aggravate gender dysphoria as well as require months to years to resume sperm production [55, 56]. In some countries, semen cryopreservation is available only through private sperm banks, which may be beyond the financial means of young people, potentially leading to inequality [15, 23, 45, 56]. A lack of awareness, knowledge, and counseling may hinder the use of fertility preservation [23].

Approximately 6 of 10 adolescents in our study did not pursue semen cryopreservation. Although we do not have information on why they opted out, we speculate that severe gender dysphoria and the request to deliver a semen sample by masturbation may play a role [18]. Procedures such as penile vibration, transrectal electrostimulation, and testicular sperm extraction (TESE) under general anesthesia are not yet available for transfeminine individuals within the Danish public healthcare system. Invasive procedures often yield a lower semen quality, but a few reports have shown that they may represent a potential alternative to masturbation. One study performed testis biopsies (mean age 13.9 years) and found sperm cells in 5 of 11 samples [46]. Other groups successfully retrieved sperm cells through TESE in 10 transfeminine adolescents (median age 14.3 years) after a median of 2 months on GnRHa [57] or by testicular biopsy in 25 individuals (median age 13.4 years), 68% of whom were successful. A testicular volume of ≥10 mL was associated with a higher success rate [58]. One group offered semen cryopreservation by masturbation or surgical retrieval [20]. A total of 78 out of 80 individuals were successful by masturbation vs 16 out of 21 by surgical procedure which also resulted in low semen quality. There is limited experience regarding fertility preservation, including invasive procedures like TESE and microscopic testicular sperm extraction (microTESE) later in life after several years of GAHT, with or without cessation of GAHT to regain spermatogenesis [12].

Our study includes all transfeminine adolescents referred to the only public national gender identity service in Denmark between 2016 through 2022 and is therefore unlikely to be affected by recruitment bias within this clinical setting. However, these data do not include adolescents with gender incongruence who do not seek public medical healthcare. In addition, access to care may be influenced by geographic distance to the service or socioeconomic factors, potentially leading to the underrepresentation of certain groups. The study has limitations including its small sample size and retrospective design with incomplete data on clinical information and lifestyle factors. We also do not have a control group from the general population matched by age, as obtaining semen samples from minors raises ethical challenges.

In conclusion, our study showed that 4 out of 10 transfeminine adolescents pursue and successfully collect semen samples for cryopreservation. However, the majority did not, suggesting that this group may miss an opportunity for future biological parenthood unless temporary cessation of GAHT or invasive fertility options prove to be successful later in life. While semen quality in this group appeared suboptimal, the underlying etiology remains unclear. Some individuals were quite young and had not yet completed puberty; they may not have reached their full potential. However, the inhibin-B/FSH balance suggests a primary spermatogenic failure rather than a centrally suppressed stimulation by gonadotropins.

Research should address whether transfeminine adolescents will have the opportunities and desire to use cryopreserved semen and achieve biological parenthood in adulthood. Further, the outcomes of alternative fertility preservation techniques should be evaluated in younger individuals seeking hormone therapy. Larger study samples may allow investigations into associations between semen quality and factors such as testis volume, pubertal staging, and lifestyle.

## Data Availability

The data underlying this article cannot be shared publicly due to national data protection regulations and for the privacy of individuals included in this study. The data will be shared upon reasonable request with the corresponding author with adherence to international data protection laws.

## Clinical Trial Information

Clinical Trials.gov, NCT06573177. Registered August 27, 2024.

## Abbreviations

BMI: body mass index
CV: coefficient of variation
FSH: follicle-stimulating hormone
GAHT: gender-affirming hormone therapy
GnRHa: gonadotropin-releasing hormone agonist
LC-MS/MS: liquid chromatography–tandem mass spectrometry
LH: luteinizing hormone
LOD: limit of detection
SDS: standard deviation score
TESE: testicular sperm extraction
WHO: World Health Organization
